# Randomised, Double Blind, Placebo-Controlled Trial of *Echinacea* Supplementation in Air Travellers

**DOI:** 10.1155/2012/417267

**Published:** 2011-12-20

**Authors:** E. Tiralongo, R. A. Lea, S. S. Wee, M. M. Hanna, L. R. Griffiths

**Affiliations:** ^1^School of Pharmacy, Griffith Health Institute, Griffith University, Gold Coast Campus, Gold Coast, QLD 4222, Australia; ^2^Genomics Research Centre, Griffith Health Institute, Griffith University, Gold Coast Campus, Gold Coast, QLD 4222, Australia

## Abstract

*Objective*. To identify whether a standardised *Echinacea* formulation is effective in the prevention of respiratory and other symptoms associated with long-haul flights. *Methods*. 175 adults participated in a randomised, double-blind placebo-controlled trial travelling back from Australia to America, Europe, or Africa for a period of 1–5 weeks on commercial flights *via* economy class. Participants took Echinacea (root extract, standardised to 4.4 mg alkylamides) or placebo tablets. Participants were surveyed before, immediately after travel, and at 4 weeks after travel regarding upper respiratory symptoms and travel-related quality of life. *Results*. Respiratory symptoms for both groups increased significantly during travel (*P* < 0.0005). However, the Echinacea group had borderline significantly lower respiratory symptom scores compared to placebo (*P* = 0.05) during travel. *Conclusions*. Supplementation with standardised Echinacea tablets, if taken before and during travel, may have preventive effects against the development of respiratory symptoms during travel involving long-haul flights.

## 1. Introduction

Intercontinental air travel can be stressful, adding extra strain on passenger's physical and psychological health [1]. Studies have investigated the occurrence of nasal dryness [2], the increased risk of developing upper respiratory disorders such as allergic rhinitis and dry coughs [3], and attracting virus- or bacteria-induced respiratory infections such as the common cold during long-haul flights [1, 4]. Almost 50% of travellers experience some kind of illness while abroad, the most common being an upper respiratory infection, typically leading to 3-day debilitation during a 14-day trip [5]. Acute respiratory tract infections like the common cold are mostly caused by rhinoviruses [6] and respiratory viral infections are also recognised as the most frequent cause of acute exacerbations of asthma and chronic obstructive pulmonary disease (COPD) [7]. 

To reduce adverse reactions to long distance flights, various interventions such as air humidification and oxygen supplementation have been trialled [8]; however, no research has investigated the possible benefit of a herbal medicine. Herbal medicines, amongst other complementary and alternative medicines, are used by over half of the population worldwide [9–11], and amongst the most widely used herbal medicines is *Echinacea*, with millions of units sold annually. 

Due to *Echinacea's* in vitro anti-inflammatory [12, 13], antiviral [14, 15] and immuno-modulating effects [16, 17], numerous clinical trials have investigated its efficacy for the treatment of the common cold. Although most studies demonstrated positive effects such as decreased severity and duration of symptoms of the common cold when *Echinacea* is given at first sign of infection [18–20], outcomes are inconsistent [21] because of the variations in treatment protocols, applied outcome measures, and *Echinacea* preparations [19, 20].

Preparations from three different species, *Echinacea angustifolia*, *Echinacea purpurea,* and *Echinacea pallida,* are generally referred to as *Echinacea* preparations; however their phytochemical profile and activity can differ significantly [22]. Clinical trials mainly support efficacy for preparations from *E. purpurea* and partially *E. angustifolia* to treat symptoms of the common cold [20] and to have an effect on stress-induced factors: hsp70 and white blood cell counts [23]. Alkylamides are considered to be part of the active constituents in *Echinacea* as their bioavailability was confirmed by human pharmacokinetic studies with alkylamides detectable in plasma of healthy volunteers 30 minutes after *Echinacea* tablet ingestion [24, 25]. They have shown to affect the immune response through cannabinoid type 2 dependent and independent pathways, modulating the production of cytokines such as TNF*α* [17] and IL-2 [26].

*Echinacea's* preventative effects for respiratory illness are still debated [19, 27, 28] and difficult for consumers to ascertain [29]. Some previous studies used artificial rhinovirus inoculation [28] or were not blinded and placebo controlled [27]. The aim of our research was to identify whether an alkylamide-standardised, bioavailable *Echinacea* formulation [23, 30] is safe and effective in the prevention of respiratory and other travel-related symptoms during travel involving long-haul flights.

## 2. Material and Methods

### 2.1. Study Design

A randomised, double-blind placebo controlled clinical trial was conducted between February 2009 and May 2010 in Australia with economy class passengers travelling back, for a period of 1 to 5 weeks, from Australia to America, Europe, or Africa on commercial flights with a flying time of 15–25 hours and less than 12-hour stopovers. The clinical trial received ethical approval from the institutional Human Research Ethics Committee (PHM0608HREC) and was registered with the Australian New Zealand Clinical Trials Registry (http://www.anzctr.org.au/ (ANZCTR 083687)).

**Figure 1:**
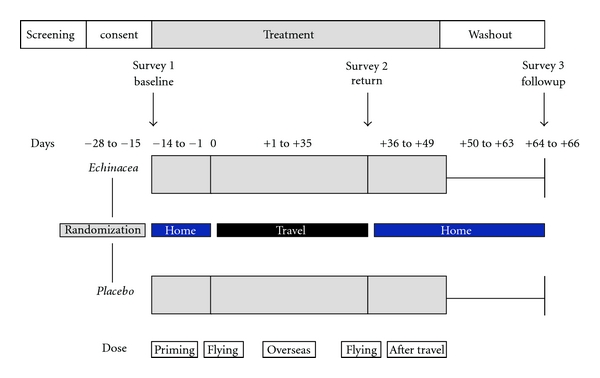
Study design of the trial for a travel time of 35 days.

Figure 1 outlines the study design for a participant travelling for 35 days. For all participants treatment would commence 14 days before flying overseas and would complete 14 days after returning to Australia. The actual treatment time varied between participants depending on their travel duration. It ranged from a minimum of 5 weeks (if 7 days/1week of travel) to 9 weeks (if 35 days/5 weeks of travel).

Each participant completed three surveys: at 14 days before travel (baseline), <1 week after travel (return), and at 4 weeks after returning from travel (followup). The surveys contained questions relating to upper respiratory symptoms, jet lag duration, headache, sleep disturbances, and cold sore covering a period of the previous 4 weeks at each individual time point (baseline, return, and followup).

### 2.2. Study Participants and Randomisation

Participants were recruited through travel agencies, radio, newspaper, and TV advertisements, and emails circulated to all staff and students at a university and a teaching hospital on the Gold Coast, Australia. Volunteers were included if they were 18–65 years of age, in good general health and suffered from no previous or current serious illness. Volunteers were excluded if they had a known plant allergy, were suffering from respiratory diseases (e.g., asthma, COPD), had any other condition that could compromise the study or the participants health (e.g., autoimmune disease, cystic fibrosis), had received flu vaccination within 20 days of starting the trial, were lactating, pregnant, or planning to become pregnant, or were on regular treatment with *Echinacea*, antibiotics, corticosteroids, antihistamines, and immunosuppressants.

One hundred and seventy-five volunteers met the inclusion criteria and were randomly assigned to trial tablets. The random allocation sequence provided by the sponsor was computer generated using a randomisation plan from http://www.randomization.com/ with randomisation in blocks of 10. A list of consecutive study numbers was generated. Treatment groups were allocated by trial staff, but the allocation was concealed by providing each participant with a number. Participants, chief investigators, and trial staff were blinded to group allocation. To confirm that blinding was effective, a subgroup of participants (*n* = 11 on placebo and *n* = 10 on *Echinacea*) were asked to speculate whether they were taking *Echinacea* or placebo. Eleven participants identified themselves correctly, whereas 12 identified themselves incorrectly. There was an even distribution of mismatches in the placebo and *Echinacea* group providing further evidence of effective randomisation.

### 2.3. Clinical Outcome Measurements and Statistical Analysis

Upper respiratory symptom-related quality of life (QoL) was measured using the questions from the 44-item Wisconsin Upper Respiratory Symptom Survey (WURSS-44), which was the primary outcome variable [31]. The WURSS-44 is a responsive, reliable, and valid instrument for evaluating QoL outcomes related to respiratory illness, measuring all significant health-related dimensions that are negatively affected by the common cold [31, 32]. It has been shown to be a more powerful instrument for assessing respiratory-related QoL then the general-health-related QoL, instrument SF-36 [32] and measurements correlate well with laboratory-assessed biomarkers [33]. The WURSS-44 includes 1 global severity item, 32 symptom-based items, 10 functional QoL items, and 1 global change item, all of which are based on 7-point Likert-type severity scales. A previous validation of the instrument showed that a cumulative score should be calculated by summing the severity scores of the first 43 items with high severity scores indicating high symptom load [31]. The median WURSS-44 score was calculated and compared for both treatment groups using the nonparametric Kolmogorov-Smirnov test for median differences in independent samples. Symptoms in our survey were assessed over the past 4 weeks rather than for the past 24 h to accommodate the travel trial setting. A minimal important difference (MID) is the term generally used to quantify the minimum amount of positive change that patients perceive and would accept an associated treatment as being beneficial or worth taking—a clinically significant effect [34]. For the WURSS-44 score, an MID of 16.7 points was determined [32]. Therefore, individuals that presented with a respiratory disorder symptoms score of 17 and above (RDS+) were compared in both groups at baseline, return, and followup. Difference in the prevalence (or proportion) of RDS+ individuals between groups at followup was statistically compared using a 2 × 2 chi-squared test of independence and the Odds Ratio. The amount of missing data was different among variables but on average was less than 10%. We analysed observed data, only that is, did not impute data or conduct missing at random analyses.

In addition 15 questions were designed and assessed as secondary outcome measures for occurrence and duration of jet lag, headache, sleep pattern, and herpes simplex sores. *t*-tests and chi-square tests were used to analyse differences between groups regarding headache, cold sore, and sleep disturbances. Results were considered significant when the *P* value was ≤0.05. All statistical analysis carried out was based on intention to treat (ITT) using the program PASW Statistics (SPSS) version 18.0.

Study participants were asked to complete a diary during the trial to record any upper respiratory symptoms, possible travel stress symptoms such as jet lag duration, headache, sleep disturbances, cold sore, headache, administration of sick dose, as well as additional health issues or disease symptoms and additional medication taken. The diary helped participants with recalling information when completing the surveys and allowed researchers to identify possible inconsistencies in data documentation. Participants were contacted by trial staff via phone a couple of days before leaving Australia and following their return to Australia, to ensure participants well-being, correct dosing, compliance, diary completion and to make final appointments. Participants were provided with an emergency phone number which they could contact 24 hours, 7 days a week, especially while being overseas.

### 2.4. Sample Size Calculation

Studies estimate the incidence of respiratory problems from 11%—in flight emergencies—based on respiratory problems [35] to 50% health-related problems with the 2nd most common cause being respiratory problems [36]. For this study it was estimated that 4 out of 10 participants in the placebo group (40%) will be RDS+ at followup compared to 20% in the *Echinacea* group, which equates to a clinically significant decreased risk of RDS (OR~2). It was also previously reported that using the WURSS-44 to assess symptomatic patients a two-armed RCT would require 92 participants in total to detect an MID as being statistically significant [32]. We concluded that a sample size of approximately 180 would yield at least 80% power to detect a treatment effect as statistically significant at the 0.05 alpha level.

### 2.5. Treatment

The *Echinacea* tablet preparation used was the commercially available *Echinacea* Premium tablets (MediHerb brand) manufactured by Integria Healthcare Pty Ltd. (AustL no. 75124) standardised to 4.4 mg alkylamides. The tablets contained 112.5 mg *Echinacea purpurea* 6 : 1 extract (equivalent to 675 mg dry root) and 150 mg *Echinacea angustifolia *4 : 1 extract (equivalent to 600 mg dry root). Phytochemical profiles for these tablets were established for previous batches [25, 30] and also determined for the batch used in this study (Table 1). Placebo tablets were manufactured to match the *Echinacea* tablets in size, excipients, and colour. Both sets of tablets were coated with a brown colour and hypromellose to make them indistinguishable. Tablets were packed in identical amber glass bottles with identical labelling. Labelling only identified the patient number.

### 2.6. Dosing

For our study medication, the manufacturer recommends for adult patients one tablet three times daily (3825 mg dry root equivalent/day) and if required an increase to two tablets three times daily. This lies within the dosage range commonly recommended for *Echinacea* formulations. For ease of adherence and compliance with the dosing schedules whilst travelling and experiencing time zone changes, dosing was undertaken twice daily, either as one tablet (priming, overseas, and after-travel dose) or two tablets during the stressful flying time (flying dose). An example of the protocol for 5 weeks of travel is given below in Table 2 (also see Figure 1 for study layout), with Day 0 being the first day of travel and Day 35 being the return day. For shorter travel periods, the period for the overseas dose between days 8 and 32 was shortened, reflecting the time the participant was spending abroad. All other dosing, before and after travel including during the washout period, remained the same for each participant.

Participants were allowed to take a sick dose (three tablets twice a day) if cold- or flu-like symptoms occurred. The sick dose could only be taken for up to 8 consecutive days or twice for 4 days during the whole travel period. Compliance was assessed by calculating the percentage of tablets taken against total tablets expected to be taken of the treatment period.

## 3. Results

**Figure 2:**
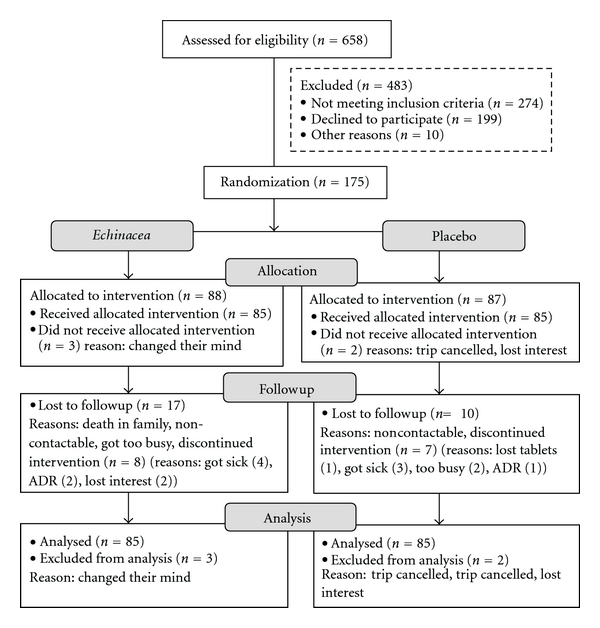
Flowchart of participants in the trial.

The flow of participants through the trial between February 2009 and May 2010 is summarised in Figure 2. Six hundred and fifty-eight people were screened, with a number deemed ineligible by inclusion criteria: plant allergy, inappropriate destination, and/or extended stopovers during travel (>12 h). Reasons for declining participation included not wanting to be on placebo, travel cancellation, tablet size, and personal circumstances. Of the 175 trial participants, 170 completed the initial survey. Thus ITT analysis was performed on 170 participants. All three completed surveys were returned by 143 participants while 27 were lost to followup.

### 3.1. Baseline Analysis

Of the 170 participants analysed, 85 were assigned *Echinacea* tablets and 85 were assigned placebo. Sixty-seven percent of participants were women.  Table 3 shows the baseline characteristics of the trial groups. On average, participants were 43 years old, weight 76 kg, had normal blood pressure, and travelled for 23 days. Travel was mostly holiday related. There were no statistically significant differences between the *Echinacea* and placebo group for the test variables at baseline (Table 3). Thus, the two treatment groups can be considered reasonably well balanced at baseline.

### 3.2. Primary Outcomes

**Figure 3:**
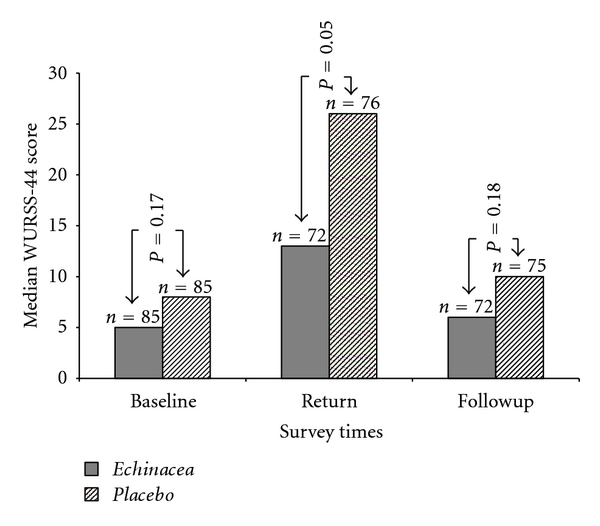
*y*-axis shows average WURSS-44 scores for each treatment group at the 3 time points surveyed (*x*-axis), *n* = 170.

Figure 3 shows the median WURSS-44 symptom scores for the placebo and *Echinacea* group at each time point. Compared to baseline (before travel), the average WURSS-44 scores for both groups increased during travel (measured retrospectively at return) (*P* < 0.0005). When comparing both groups with each other at each individual time point, the WURSS-44 scores did not differ significantly before travel (baseline) (*P* = 0.17) and during the 4 weeks after travel (measured retrospectively at followup) (*P* = 0.18). However, at during travel (measured retrospectively at return), the placebo group had significantly higher WURSS-44 scores on average compared to the *Echinacea* group (26 versus 13, *P* = 0.05).

When comparing the percentage of participants considering themselves to be affected by respiratory illness (score > 17), there was no significant difference between both groups at baseline (before travel) (*P* = 0.19). However, the results from the survey completed immediately after return from travel showed a significantly reduced percentage of RDS+ affected participants in the *Echinacea* group compared to placebo (43% versus 57%, *P* = 0.05) during travel. This difference was further substantiated during the 4 weeks after travel (survey at followup) where there was a significantly lower percentage of illness in the *Echinacea-*treated group compared to placebo (i.e., 25% versus 39%) which corresponds to ~50% relative reduction (*P* = 0.03). This implies that patients will be 50% less likely to suffer respiratory disorder symptoms scores of 17 and above (RDS+), which they consider treatment worthy.

### 3.3. Secondary Outcomes

Given *Echinacea*'s anti-inflammatory and antiviral effects we also investigated whether this herbal medicine could be beneficial in the prevention and treatment of headache and cold sores, respectively. However, there was no significant difference between the placebo and *Echinacea* group with regards to sleep disturbances and the prevalence of headache and cold sores at any of the time points (data not shown).

### 3.4. Compliance and Adverse Events

Treatment compliance was high in both groups with no significant difference in their compliance to the study medication (*Echinacea* = 92.5% versus Placebo = 95%, *P* = 0.49). Similarly, the use of sick doses was not significantly different between both groups (*Echinacea* = 47% versus Placebo = 53%, *P* = 0.77).

Overall, treatment was well tolerated. Adverse events such as (i) vomiting and headache, (ii) heartburn, and (iii) diarrhoea were reported by only 3 participants, respectively, but a causal relationship between *Echinacea* and the events could not be established. The participant who reported headache and vomiting was later unblinded as taking placebo tablets and the participant who reported heartburn and diarrhoea were later unblinded as taking *Echinacea* tablets. However, the participants who reported heartburn was also taking aspirin and several other medicines such as sleeping tablets.

However, two participants, who were later identified as taking *Echinacea*, reported symptoms that were regarded as potential adverse effects from *Echinacea*. Both participants reported tingling and burning of the tongue and mouth immediately after taking tablets. While one participant stopped the tablets immediately and the symptoms disappeared within 24 h, the other participant continued the trial medication for over 3 weeks while symptoms worsened (sore throat, achy head, swollen legs, fever, rash, redness and itchiness on feet and legs, and a general feeling of being unwell). Following the cessation of the trial medication, symptoms disappeared within 1–21 days depending on severity and affected areas. A full blood count was normal for all parameters 3 weeks after stopping the tablets.

## 4. Discussion

In this study, both placebo and *Echinacea* groups experienced respiratory illness during travel, which was indicated by raised upper respiratory symptom scores and a higher percentage of respiratory disease symptom-affected participants. This is an expected outcome as previous research has reported increased medical issues including respiratory symptoms associated with commercial flights independent of aircraft types [1]. Studies estimate the incidence of respiratory problems between 11% as in flight emergencies based on respiratory problems [35] and 50% as health-related problems with the 2nd most common cause being respiratory problems [36]. Importantly, this study provides some indication that, at return from travel, participants using *Echinacea* displayed a lower respiratory symptom score and the overall percentage of participants affected by respiratory disease symptoms was marginally lower in the *Echinacea* group compared to placebo. This suggests that the *Echinacea* treatment may had a protective effect against the development of respiratory symptoms during the period of travel.

This study is the first prevention trial for *Echinacea* use that employs the recommended WURSS-44 as an outcome measure for respiratory-illness-related QoL. In contrast to a recent prevention trial [27] it describes a well-powered, high-quality, placebo-controlled study. Moreover, it uses a common scenario of travel as a risk factor of attracting respiratory illness rather than artificial virus inoculation [28].

The reported discrepancy in previous clinical trial results for *Echinacea* is attributed to variations in applied outcome measures, treatment protocols, and, most importantly, varying *Echinacea* preparations [19, 20]. Specifically, the evaluation of herbal study medications is essential for the interpretation of clinical trial results [37], and the evidence of efficacy (and safety) for herbal medicines should be considered to be extract specific [38]. This study utilised a standardised *Echinacea* formulation for which a phytochemical profile and pharmacokinetic data exist [25] and which has been marketed for reducing the incidence and symptoms of cold- and flu-like symptoms in Australia, New Zealand, the UK, the USA, Canada, and South Africa.

Similar to the three previously mentioned prevention and inoculation trials where they used 7–14-day pre- and 5-day postinoculation treatment [18, 28], participants in this study were treated 14 days prior to travel, during travel, and 14 days after travel, thus using a similar pre-, but slightly longer posttreatment. During travel (measured at return from travel) the differences between the *Echinacea* and placebo group were borderline significant for symptom scores and the number of participants who suffered from treatment worthy respiratory disorder symptoms (scores of 17 and above (RDS+)). This difference was further substantiated during the 4 weeks after travel (measured at follow-up), as a ~50% relative reduction of illness in the *Echinacea* treated group compared to placebo was observed. Our results suggest that 1 in 2 patients who suffer RDS+ would have benefited from *Echinacea* supplementation during travel.

Of note, an influence of travel duration on the results is excluded as no significant difference was found between both treatment groups.

Preparations and dosing schedules used in previous treatment and prevention trials vary widely [18, 19, 28]. Our results indicate that the dosing chosen in this study will be sufficient to have a beneficial effect on respiratory health while travelling.

The WURSS-44 originally developed for the assessment of patients affected by cold and flu [31] was strongly recommended for *Echinacea* trials [19] and was therefore utilised in this study. Whether *Echinacea* will benefit general QoL is still debated [39], and whether it affects headache, sleep pattern, jet lag, and herpes simplex duration should be investigated by future studies using appropriate outcome measures.

Numerous human trials have found that *Echinacea* is well tolerated with a slight risk of transient, reversible adverse events involving mainly gastrointestinal upsets and rashes [20, 40]. In rare cases, *Echinacea* can be associated with allergic reactions that may be severe or exacerbate asthma [40]. Hence, plant allergy and asthma became exclusion criteria in this trial. As expected, *Echinacea* was generally well tolerated in this trial. We observed only two adverse events which displayed a highly likely relationship to *Echinacea* as they classified as allergic reactions and mimic description of allergic reactions previously described for *Echinacea* [40]. Importantly, both participants recalled mild reactions to some weeds in the past. Given these findings, it should be considered to alert consumers and patients to the possibility of allergic reactions to *Echinacea* if they are allergic to plants in general. It is also important to note that one participant had taken other *Echinacea* products previously without experiencing adverse effects. This again highlights the importance of well-characterized and standardized herbal products to be able to compare efficacy and safety outcomes.

As with all RCTs, this study had several limitations. Whilst diaries were used to document events during participants' travel and help with the recollection of dosing, symptoms, and compliance, a certain amount of recall bias has to be expected when the surveys were completed at return and followup. This study used travel including long-haul intercontinental flights, time zone, and climate change as a model to see whether *Echinacea* is effective in preventing upper respiratory symptoms. Whether these findings can be generalised for other populations is debatable. In addition, the appropriateness of *Echinacea* use needs to be established for each individual traveller, especially those suffering from respiratory diseases (e.g., COPD, asthma, pneumonia) or immune disorders as they belong to the group of people who were excluded from this trial.

## 5. Conclusion

Although respiratory symptoms for both groups increased significantly during travel periods associated with long-haul flights, the increase of these symptoms for the *Echinacea* group was significantly lower than for the placebo group. This highlights a beneficial effect from *Echinacea* supplementation in adults if tablets contain 4.4 mg alkylamides from *E. purpurea *and* E. angustifolia* and are taken 14 days before and during travel. The incidence of adverse effects was low and may be predicted by thoroughly assessing the patient's medical history for plant allergies and counselling on the above-mentioned signs and symptoms of allergy thus enabling consumers to stop *Echinacea* formulations at the onset of an allergic response.

## Figures and Tables

**Table 1:** Alkylamide content per *Echinacea* tablet used as trial medication.

Alkylamide name	MW	Structure	mg/tablet^a^
(2E/Z,4Z/E)-N-(2-Methylpropyl)undeca-2,4-diene-8,10-diynamide	229	Diene	0.247
(2E,4Z)-N-(2-Methylpropyl)dodeca-2,4-diene-8,10-diynamide	243	Diene	0.505
(2E,4E,8Z/E,10Z/E)-N-(2-Methylpropyl)dodeca-2,4,8,10-tetraenamide	247	Diene	1.504
(2E,4E,8Z)-N-(2-Methylpropyl)dodeca-2,4,8-trienamide	249	Diene	0.078
(2E,4E)-N-(2-Methylpropyl)dodeca-2,4-dienamide	251	Diene	0.166
(2E,4Z)-N-(2-Methylbutyl)dodeca-2,4-diene-8,10-diynamide	257	Diene	0.130
(2E/Z)-N-(2-Methylpropyl)undec-2-ene-8,10-diynamide	231	Monoene	0.699
(2E)-N-(2-Methylpropyl)dodec-2-ene-8,10-diynamide	245	Monoene	0.452
(2E,7Z)-N-(2-Methylpropyl)trideca-2,7-diene-10,12-diynamide	257	Monoene	0.081
(2E0-N-(2-Methylbutyl)dodec-2-ene-8,10-diynamide	259	Monoene	0.186
(2E,9Z)-N-(2-Methylpropyl)pentadeca-2,9-diene-12,14-diynamide	285	Monoene	0.330
(2E,9Z)-N-(2-Methylpropyl)hexadeca-2,9-diene-12,14-diynamide	299	Monoene	0.041

Total			4.419

^a^ Alkylamide concentrations were determined by liquid chromatography mass spectrometry using a Shimadzu HPLC system coupled to a Shimadzu 2010EV single quadrupole mass spectrometer operating with an APCI interface as described for a previous batch [25].

**Table 2:** Treatment protocol used for 35 days of travel.^a^

Days	Protocol	Dosage
−14 to −3	Priming dose	One tablet twice a day
−2 to +7	Flying dose	Two tablets twice a day
+8 to +32	Overseas dose	One tablet twice a day
+33 to +42	Flying dose	Two tablets twice a day
+43 to +49	After-travel dose	One tablet twice a day

^a^Also see Figure 1 for study layout.

**Table 3:** Comparison of demographics and outcome measures of trial groups at baseline (before travel).

Variable	Total (*n* = 170)	*Echinacea* (*n* = 85)	Placebo (*n* = 85)	*P* value^a^
Age in years-mean (SD)	43 (14)	44 (13)	42 (14)	0.21
Females (%)	113 (67)	62 (55)	51 (45)	0.10
Weight in kg (SD)	75.6 (17.8)	75.0 (16.8)	76.0 (19.2)	0.48
BMI (SD)	26.0 (5.3)	25.8 (5.0)	26.3 (5.6)	0.55
Diastolic blood pressure (SD)	86.7 (8.4)	85.8 (8.7)	88.1 (7.9)	0.10
Systolic blood pressure (SD)	110.6 (11.8)	111.1 (11.9)	109.7 (13.5)	0.48
Duration of travel in days (SD)	23 (8)	23 (8)	22 (8)	0.45

^ b^RDS + (%)	39 (23)	17 (20)	22 (26)	0.38
^ c^Median WURSS-44 score	7	5	8	0.17

Cold sore (%)	63 (37)	31 (36)	32 (38)	0.87
Sleep difficulties (%)	40 (24)	21 (25)	19 (22)	0.72
Headache sufferer (%)	66 (39)	32 (38)	34 (40)	0.85

^a^All *P* values are two-tailed comparing *Echinacea* to placebo. Values are either means with standard deviations (SD) or frequencies with percentages (%). ^b^RDS+ indicates a Respiratory Disease Symptoms score > 17. ^c^Median WURSS-44 score refers to the median Wisconsin Upper Respiratory Symptom score.
